# Is Face and Mask Touching a Real Struggle During the COVID-19 Pandemic? A Prospective Study Among Medical Students

**DOI:** 10.3389/fmed.2021.663873

**Published:** 2021-07-29

**Authors:** Aleksandra A. Stefaniak, Piotr K. Krajewski, Rafał Białynicki-Birula, Danuta Nowicka, Jacek C. Szepietowski

**Affiliations:** Department of Dermatology, Venereology and Allergology, Wroclaw Medical University, Wroclaw, Poland

**Keywords:** face-touching, mask-touching, COVID-19, facemasks, COVID-19 spreading

## Abstract

There are limited data in the literature on the frequency of face- and mask-touching behavior as a potential vector for the self-inoculation and transmission of the novel coronavirus. In this prospective study, we assessed the facial touching behavior of 204 medical students. One hundred thirty-four subjects (65.68%) during the 15-min observation at least once touched the area of the mask (38.23%), eyes (38.23%), or other parts of the facial zone (49.02%). The mean number of touches was 11.98 ± 16.33 per hour. The results of our study reveal that there is no significant association between mask wearing and gender; however, there might be a tendency for people with eyeglasses to touch the area near the eyes more often.

## Introduction

COVID-19 is an infectious disease, which is mainly transmitted through aerial droplets and direct contact with contaminated surfaces and mucosal membranes (1–4). The use of face coverings (including masks) can prevent the introduction of the virus through the expulsion of airborne droplets to the environment (2–5). When the novel coronavirus was detected as a factor causing a new respiratory disease, the Chinese government asked members of the public to wear face masks in a public space, a measure that is now thought to have aided in controlling the pandemic (2–4, 6). Following this regulation, the Centers for Disease Control and Prevention in the USA made a recommendation for the use of face masks in public, although this was not mandatory. Several countries in Europe have also adopted laws requiring citizens to wear face masks in public (3, 4, 7). There is an increasing acceptance of the benefits of the use of masks and face coverings by the general population in public settings (3, 4, 7). In a growing number of societies, mask wearing has become a symbol of awareness and participation in the collective effort to fight the pandemic.

However, several concerns have been raised by the researchers regarding wearing a mask. It was proven that the risk of respiratory tract infection due to hand touches to target facial membranes (the conjunctivae of the eyes, the lips, and the mucous membranes of the nostrils) depends also on the rate of contact (number per unit time) with these targets. It is possible that mask wearing might affect the hand–face contact. On the one hand, the mask protects the area of the lips and nostrils from being touched. On the other hand, they might increase the contact in the mask area because of the necessity to adjust the mask during wearing and mask-induced itching (8).

This study aimed to assess the frequency of face touching in subjects wearing face masks and examine the possible factors that may influence this behavior.

## Materials and Methods

In this prospective study, we have approached 204 consecutive subjects (142 women and 62 men) in the university clinic settings—all subjects were fourth-year medical students wearing surgical masks. Four investigators were examining the groups. In one investigated group, there were three to nine subjects at one time. The group was observed each time for 15 min. The observer made no verbal contact with the participants and the groups did not know what the purpose of the observation was. The results were multiplied by a factor of four to achieve in result the number of touches per hour. The following criteria that may influence face touching were taken into consideration: gender (male/female) and whether the subject was wearing eyeglasses. The area of contact was divided as follows: mask, eyes, and other parts of the facial zone. The observers used a checklist to record the number of times when each observed person touched the face in the abovementioned areas. The study was performed in December 2020, when mask wearing in public institutions, such as hospitals, was obligatory by Polish law.

Statistical analysis was performed using Statistica v. 12 (StatSoft Kraków). The minimum, maximum, mean, and standard deviation values were calculated. Analyzed quantitative variables were compared using the Mann–Whitney *U* test and Spearman and Pearson correlations, while for qualitative data test, χ^2^ was used. A two-sided *p*-value ≤ 0.05 was considered to be statistically significant.

## Results

During the 15-min observation, 134 subjects (65.68%) touched the area of the mask (38.23%), eyes (38.23%), or other parts of the facial zone (49.02%) at least once. None of the subjects used the protective visor. The mean number of touches in the whole analyzed areas was 11.98 ± 16.33 per hour, and the maximum number of touches in one person was 100. The mean number of touches in the mask area was 4.96 ± 10.1 per hour, in the eye area it was 1.96 ± 4.1 per hour, and in the other parts of the facial zone it was 5.08 ± 7.64 per hour. There were no statistically significant (*p* > 0.05) differences between the number of touches in different areas.

Concerning gender, there were no statistically significant differences in the analyzed groups (Table 1).

**Table 1:** The scores and dependencies on selected clinical data.

	**Women (*n* = 142)**	**Men (*n* = 62)**	***P***
Total number of touches per hour (*n*), mean ± SD (range)	13.15 ± 18.05 (0–100)	9.29 ± 11.14 (0–48)	NS
Mask area touches per hour (*n*), mean ± SD (range)	5.1 ± 11.0 (0–76)	4.64 ± 7.74 (0–36)	NS
Eye area touches per hour (*n*), mean ± SD (range)	2.17 ± 4.46 (0–16)	1.48 ± 3.09 (0–12)	NS
Other parts of the facial zone touches per hour (*n*), mean ± SD (range)	5.89 ± 8.52 (0–44)	3.21 ± 4.55 (0–4)	NS

*SD, standard deviation; NS, not significant*.

Another variable analyzed by our group was the usage of eyeglasses. Seventy subjects (34.31%) in our group were wearing eyeglasses during the examination: 46 women and 24 men. There were no statistical differences between these two groups concerning face touching (detailed data not shown). However, the number of subjects with eyeglasses who have touched the eye area at least once during the observation period was higher compared to the number of subjects without eyeglasses touching this area and almost reached statistical significance (*p* = 0.05).

## Discussion

The results of our study revealed that there is no significant association between mask wearing and gender; however, there might be a tendency for people with eyeglasses to touch the area near the eyes more often. Sixty-five percent of the subjects have touched their face area at least once during the 15-min observation. This number is much lower than that reported previously by Shiraly et al. (9) (Table 2). In their study conducted in Iran, trained observers watched the participants in public spaces (parks, banks, outpatient clinics, and bus stations) for 15–30 min for each person. They approached 1,000 participants, including 568 wearing masks. Although in their study, more than 90% of subjects touched their face area during the observation, the number of touches per hour was lower compared to our study. Our subjects were observed for 15 min and subjects in the Iranian study were observed for 15 min up to 30 min, which may explain the higher percentage of subjects who made at least one face touch. However, the difference in the number of touches per hour remains unclear, especially concerning the fact that our subjects were students from medical and dentistry faculties, who were extensively informed about the ways of the novel coronavirus spreading, while the comparative study was made in the first few days after lifting quarantine restrictions in Iran. The authors of the study underline the fact that in people without a mask, there were significantly more face touches regardless of the zone compared with those wearing a mask (*p* < 0.001). Moreover, subjects without masks touched their mucosal zone more frequently than mask wearers (5.5 vs. 1.9 times per hour, *p* < 0.001).

**Table 2:** Comparison among the series.

**Authors (year)**	**Subjects (*n*)**	**Percent of subjects who made at least one face touch (%)**	**Number of face touches per hour (** ***n*** **), mean** **±** **SD (range)**	
			**With mask**	**Without mask**
Shiraly et al. (9)	1,000 (568 with and 432 without masks)	92%	8 ± 5 (n.d.–n.d.)	11 ± 6 (n.d.–n.d.)
Lucas et al. (10)	40 (24 with and 16 without masks)	Not revealed	5.4 ± n.d. (2.3–17.8)	20 ± n.d. (12.8–22.9)
Current study	204 subjects with masks	65.68%	11.98 ± 16.33 (0–100)	Not studied

*SD, standard deviation; n.d., no data*.

Interestingly, the number of face touches in our study was higher compared to the study conducted by Lucas et al. (10) in California (Table 2). They reached a total of 40 subjects (physicians in hospital settings)−24 were wearing masks most of the time and 16 were not in six observation sessions. The duration of each observation session ranged from 20 person-minutes to 120 person-minutes. The total face touches were 83 (time period not revealed), with 29 face touches in healthcare professionals with masks and 54 face touches in subjects not wearing a mask. The median face touches per hour for healthcare providers with masks was 5.4 (2.3–17.8) compared to 20 (12.8–22.9) in those without a mask (the significance was not revealed).

Tao et al. reached and analyzed surveillance videos recorded on the buses before and after the COVID-19 outbreak (11). All facial touching events were counted in 30-min intervals by two observers in each video. Thirty-one passengers with no masks touched their faces 71 times in total (0–11 times, median = 1.0, 95% CI: 0–3.0 times), while 30 passengers with masks touched their face 66 times in total (0–10 times, median = 1.0, 95% CI: 0–2.0 times), including touching or adjusting their masks 54 times (0–9 times, median = 1.0, 95% CI: 0–2.0 times). There was no significant difference in total facial touching events between the analyzed groups (*p* > 0.05). The second observation was made on the group having been subjected to high-intensity public education programs explaining the role of facial touching in the spread of COVID-19. We may relate this observation to our own made on medical students. However, the researchers underlined the fact that the use of masks did not increase (or decrease) the total number of facial touches.

It was documented previously that the health-related attitudes, behaviors, and practices differ between medical and non-medical students (12). Moreover, it was observed that medical students used face masks more commonly, for longer time periods, and less frequently reused the disposable masks (13). This group has been studied previously, before pandemic settings, in the area of face touching—Kwok et al. (14) in Australia observed 26 medical students for face touches. Altogether, the students made 2,346 touches to the face over 240 min (an average participant made 23 face touches per hour); 56% of all of the touches were in the mucosal area. The mask-induced itch may occur in 14.9–51.4% of people wearing face masks (15–17). Nearly 20% of the affected individuals in the online self-assessment surveys reported scratching their face without removing the mask, about 10% took their masks off and then scratched the skin, and only 6.2% took the mask off and did not use it for some time (15). Face touching is an instinctive behavior that helps to overcome stress, regulate emotions, and stimulate memory (18). That is why there might be a possibility that despite the educational process and knowledge of the possible result of the inoculation of the virus and subsequent infection, the subjects are not able to overcome the unconscious primary desire to touch their face.

We are aware of the limitations of the study. To achieve the number of touches per hour, the observed results were multiplied by a factor of four, which may affect the outcome of the study. Moreover, this study population was a relatively homogeneous group, which makes it more difficult to generalize to other patient populations.

In conclusion, the number of face touches per hour seems to be independent of the several analyzed factors in our study—gender and usage of eyeglasses, although there might be a tendency for more frequent eye area contact in people wearing eyeglasses. Moreover, based on the previous literature, it seems that face touching does not correlate with mask wearing or with the level of education about the virus spread. These may suggest that the face touching behavior tendency depends on other factors, such as personal beliefs and the person's upbringing. A prospective study on the potential role of these social factors would be appreciated.

## Data Availability Statement

The data that support the findings of this study are available on request from the corresponding author. The data are not publicly available due to privacy or ethical restrictions.

## Ethics Statement

The authors confirm that the ethical policies of the journal, as noted on the journal's author guidelines page, have been adhered to and done under the department statutory ethical committee approval (KB - 520/2018).

## Author Contributions

AS, PK, RB-B, DN, and JS contributed to conception and design of the study. AS, PK, RB-B, and DN were investigating the subjects. AS organized the database and wrote the first draft of the manuscript. PK performed the statistical analysis and wrote sections of the manuscript. JS was supervising the study during all stages. All authors contributed to manuscript revision, read, and approved the submitted version.

## Conflict of Interest

The authors declare that the research was conducted in the absence of any commercial or financial relationships that could be construed as a potential conflict of interest.

## Publisher's Note

All claims expressed in this article are solely those of the authors and do not necessarily represent those of their affiliated organizations, or those of the publisher, the editors and the reviewers. Any product that may be evaluated in this article, or claim that may be made by its manufacturer, is not guaranteed or endorsed by the publisher.
